# *CXCR1 *and *SLC11A1 *polymorphisms affect susceptibility to cutaneous leishmaniasis in Brazil: a case-control and family-based study

**DOI:** 10.1186/1471-2350-11-10

**Published:** 2010-01-20

**Authors:** Léa Castellucci, Sarra E Jamieson, E Nancy Miller, Eliane Menezes, Joyce Oliveira, Andrea Magalhães, Luiz Henrique Guimarães, Marcus Lessa, Amélia Ribeiro de Jesus, Edgar M Carvalho, Jenefer M Blackwell

**Affiliations:** 1 Federal University of Bahia, Salvador, Brazil; 2 Cambridge Institute for Medical Research and Department of Medicine, University of Cambridge School of Clinical Medicine, Cambridge, UK; 3 Telethon Institute for Child Health Research, Centre for Child Health Research, The University of Western Australia, Subiaco, Western Australia, Australia; 4 Instituto de Investigação em Imunologia, São Paulo, Brazil; 5 Universidade Federal de Sergipe - Aracaju, Brazil

## Abstract

**Background:**

*L. braziliensis *causes cutaneous (CL) and mucosal (ML) leishmaniasis. Wound healing neutrophil (PMN) and macrophage responses made following the bite of the vector sand fly contribute to disease progression in mice. To look at the interplay between PMN and macrophages in disease progression in humans we asked whether polymorphisms at genes that regulate their infiltration or function are associated with different clinical phenotypes. Specifically, *CXCR1 (IL8RA) *and *CXCR2 (IL8RB) *are receptors for chemokines that attract PMN to inflammatory sites. They lie 30-260 kb upstream of *SLC11A1*, a gene known primarily for its role in regulating macrophage activation, resistance to leishmaniasis, and wound healing responses in mice, but also known to be expressed in PMN, macrophages and dendritic cells.

**Methods:**

Polymorphic variants at *CXCR1*, *CXCR2 *and *SLC11A1 *were analysed using Taqman or ABI fragment separation technologies in cases (60 CL; 60 ML), unrelated controls (n = 120), and multicase families (104 nuclear families; 88 ML, 250 CL cases) from Brazil. Logistic regression analysis, family-based association testing (FBAT) and haplotype analysis (TRANSMIT) were performed.

**Results:**

Case-control analysis showed association between the common C allele (OR 2.38; 95% CI 1.23-4.57; P = 0.009) of CXCR1_rs2854386 and CL, supported by family-based (FBAT; Z score 2.002; P = 0.045) analysis (104 nuclear families; 88 ML, 250 CL cases). ML associated with the rarer G allele (Z score 1.999; P = 0.046). CL associated with a 3' insertion/deletion polymorphism at SLC11A1 (Z score 2.549; P = 0.011).

**Conclusions:**

The study supports roles for *CXCR1 *and *SLC11A1 *in the outcome of *L. braziliensis *infection in humans. Slc11a1 does not influence cutaneous lesion development following needle injection of *Leishmania *in mice, suggesting that its role here might relate to the action of PMN, macrophage and/or dendritic cells in the wound healing response to the sand fly bite. Together with the *CXCR1 *association, the data are consistent with hypotheses relating to the possible role of PMN in initiation of a lesion following the delivery of parasites via the sand fly bite. Association of ML with the rare derived G allele suggests that PMN also have an important positive role to play in preventing this form of the disease.

## Background

Leishmania infection is associated with a broad spectrum of clinical phenotypes and many studies have demonstrated that host genetic factors play a part in determining the outcome of infection (reviewed [1-3]). *L. braziliensis *infection causes cutaneous leishmaniasis (CL) with prolonged time to lesion healing. Pro-inflammatory cytokines, including tumor necrosis factor (TNF) and interferon-γ (IFN-γ), and macrophage activation are important in eventual self-healing, but an exaggerated response is associated with mucosal leishmaniasis (ML) [4,5]. Pro-inflammatory responses elicited by polymorphonuclear neutrophils (PMN) as part of the wound healing response to the bite of the sand fly vector are important in initiation of lesion development in mice [6]. It has been hypothesized [7] that differences in the ability of macrophages and dendritic cells from different inbred mouse strains to respond to apoptotic versus necrotic PMN arising during the wound healing response to an infected sand fly bite determines disease progression. The arrival and maintenance of infiltrating cells at bite sites is thought to be mediated by sand fly-derived factors that either mimic a tissue damage signal or activate chemokine/chemokine receptor pathways [8-10]. Expression patterns for chemokines have been associated with the evolution of large and small lesions in mice following *L. braziliensis *infection, influenced by both the strain of parasite [10] and the mouse genetic background [8].

One way to look at the interplay between PMN and macrophages in disease progression in humans is to determine whether polymorphisms at genes that regulate their infiltration or function are associated with different clinical phenotypes following infection with *Leishmania *spp. *CXCR1 (IL8RA) *and *CXCR2 (IL8RB) *are receptors for chemokines that attract PMN to inflammatory sites. They lie on human Chromosome 2q25 230-260 kb upstream of *SLC11A1*, a gene that regulates macrophage activation and resistance to visceral leishmaniasis (reviewed reference [11]) as well as wound healing responses in mice [12]. Here we report on a small case-control study, underpinned by family-based analysis, which provides evidence for separate roles for *CXCR1 *and *SLC11A1 *in determining susceptibility to leishmaniasis caused by *L. braziliensis *in Brazil.

## Methods

The study was conducted in the area of Corte de Pedra, Bahia, Brazil, where *L. braziliensis *is endemic. Corte de Pedra is in a region of rural rain forest, where agriculture underpins the local economy. Around 3300 subjects were interviewed during 4 years to select the study population. For this genetics study, both case-control and family-based cohorts were studied. Index cases of ML were ascertained from medical records of the Corte de Pedra Public Health Post. The case definition of ML is a characteristic mucosal lesion with either parasitological confirmation or two of the three following criteria: positive delayed-type hypersensitivity test (DTH), positive leishmania serology, and a histopathology suggestive of leishmaniasis. All cases in the current study also responded to antileishmanial therapy. The families and neighborhoods of these ML patients were revisited, to establish the study population. CL is defined as the presence of a single chronic ulcerative lesion at a skin site without evidence of mucosal involvement, and without evidence of dissemination to 10 or more sites (disseminated leishmaniasis), also confirmed by detection of parasites or two of the three criteria listed above. Past cases that have been treated in the health post of Corte de Pedra have their diagnoses confirmed by medical records using the same criteria defined above, and all cases were examined for detection of a characteristic well delimited scar. Informed consent was obtained from all the participants, and the research was approved by the ethical committee of the Hospital Universitário Professor Edgard Santos, Salvador, Brazil. Demographical, epidemiological and phenotype characteristics of these subjects were previously described in full [13].

DNA was available from 60 ML index cases (47 males: 13 females; mean age ± SD = 40 ± 17 years), 60 age- and gender-matched unrelated CL cases (47 males: 13 females; mean age ± SD = 41 ± 17.8 years), 60 age- and sex-matched unrelated individuals (47 males: 13 females; mean age ± SD = 38 ± 18 years) positive for the leishmanin delayed hypersensitivity skin-test response (DTH^+^) and with no current or previous history of CL or ML disease, and 60 unrelated neighbourhood controls (47 males: 13 females; mean age ± SD = 40 ± 18 years). These neighborhood controls (NC) also had no clinical history of disease or leishmaniasis scars, but their leishmanin skin test status was unknown. For some of the case-control analyses the two patient groups, CL and ML, were analysed together to determine susceptibility to *L. braziliensis per se*, and the DTH^+ ^and NC groups pooled as the unaffected controls. This 120 cases compared to 120 controls analysis had ≥65% power to detect an odds ratio ≥2 at *P *= 0.01 for markers with minor allele frequency (MAF) ≥0.2. The smaller comparison of 60 cases compared to 60 controls had ≥55% power to detect an odds ratio ≥2 at *P *= 0.05 for markers with minor allele frequency (MAF) ≥0.2. The 60 ML index cases were also used to ascertain a total of 67 multi-case leishmaniasis (mixed for CL and ML) pedigrees (104 nuclear families), providing a total of 88 ML cases (i.e. 28 additional cases; 15 of the 88 cases had no observable scar for prior CL disease) and 250 CL cases (exclusive of the 60 CL cases used in the case-control study. Thus the family study is used to validate associations for ML seen in the case-control analysis, while the CL sample in families is independent and can be used to replicate observations made in the case-control analysis. TDT power approximations [14] show that the 250 CL cases in 104 nuclear families had ≥85% power to detect an odds ratio ≥2 at *P *= 0.01 for markers with minor allele frequency ≥0.2. It is possible that some CL cases in our study could progress to ML disease at a later date. Epidemiological studies show that this will affect <4% of CL patients [15], thus representing a small reduction in the power of our study to detect CL-specific genetic effects. Full demographic and epidemiological information in relation to multi-case families used in this study have been presented elsewhere [16].

Genotyping was performed in Cambridge using Taqman or ABI fragment separation technologies for polymorphisms at *CXCR1, CXCR2 *and *SLC11A1 *as presented in Table 1. Genotype data are available on request for meta-analysis. All were in Hardy Weinberg Equilibrium in genetically unrelated founders of the families, and in the unrelated neighborhood controls (data not shown). Case-control data were analysed using logistic regression analysis. PEDCHECK [17] was used to determine Mendelian inconsistencies within families. Inconsistencies due to mis-paternities had already been removed as part of previous studies [13] (L. Castellucci, unpublished PhD thesis). Mendelian inconsistencies for individual markers in this study were due to errors (<2%) that occur in calling Taqman genotyping and were set to zero for analysis. Family-based allelic association tests based on the transmission disequilibrium test (TDT) but generalized to allow analysis under additive and dominant models of inheritance were performed within FBAT [18,19] under the null hypothesis of "no linkage and no association". Unaffected members of the pedigrees were included in the study, contributing genotype information to increase statistical power of the FBAT analysis, especially for families with missing parents. Family-based haplotype TDT was performed using TRANSMIT [20]. Nominal *P*-values are presented throughout, i.e. without correction for multiple testing.

**Table 1:** Information on the polymorphic markers genotyped for *CXCR1*, *CXCR2 *and *SLC11A1*.

Gene/Marker	Physical Position (bp)	Alleles^1^	MAF	Caucasian	Asian	African
CXCR2_rs4674259 (5' UTR)	218699250	T>C	0.388	0.517	0.341	0.883

CXCR1_rs2854386 3' region	218735747	C>G	0.218	0.058	0.100	0.425

CXCR1_rs2234671 Exon 1 S276T	218737353	C>G	0.194	0.058	0.100	0.317

CXCR1_rs3138060 Intron 1	218739745	G>C	0.142	0.058	0.102	0.129

SLC11A1_rs7573065 (-237 bp 5' UTR)	218954951	C>T	0.090^3^	-	-	0^4^

SLC11A1_rs2276631 (274^2^; exon 3, Phe66Phe)	218957257	C>T	0.289	0.224	0.102	0.127

SLC11A1_rs3731865 (469+14G/C^2^; intron 4)	218958247	G>C	0.227	0.27^2^	0.08^2^	0.120^4^

SLC11A1_rs17221959 (823^2^; exon 8, Gly249Gly)	218960874	C>T	0.266	0.02^2^	0.15^2^	0.310^4^

SLC11A1_rs2279015 (1465-85G/A^2^; intron 13)	218967514	C>T	0.379	0.342	0.307	0.942

SLC11A1_rs17235409 (1703^2^; exon 15, D543N)	218967976	G>A	0.089^3^	0.01^2^	0.18^2^	0.120^4^

SLC11A1_ rs17235416 (1729+55del4^2^; 3' UTR TGTG IN/DEL)	218968058	IN/DEL	0.176	0.01^2^	0.18^2^	0.188^4^

Physical positions of markers are given according to Build 36.3 of the human genome. Where available, allele frequencies for the minor allele (MAF) in Brazil are shown for Caucasian, Asian and African populations (Hapmap CEU, JPT and YRI unless otherwise referenced). ^1 ^Major>minor alleles for this Brazilian population; ^2^[21], bp positions of variants relative to an arbitrary site 76 bp upstream of the methionine start codon; ^3^Allele frequency too low to be taken forward in the association analyses; ^4^[31].

## Results

The case-control groups had similar demographics, including age, duration of residence in the endemic area, housing and main occupation. The environmental exposures surveyed were also similar between the two family cohorts, except for house distance from the forest that was different between the CL (265.2 meters) and ML groups (144.1 meters) (*P *= 0.04, unpaired *t *test), but not between the ML group and the NC or DTH-positive groups, as fully described elsewhere [13].

**Figure 1:**
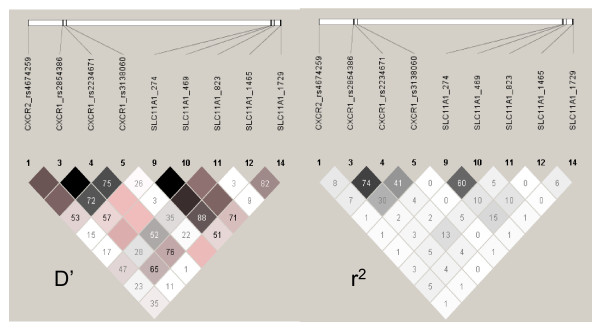
**Haploview analysis for D' and r^2 ^pairwise measures of LD between *CXCR2, CXCR1 *and *SLC11A1 *SNPs and IN/DEL in family founders from the study population in Brazil**. D' values and confidence levels (LOD) are represented as black for D' = 1, LOD>2; shades of pink for high D', LOD<2; white for D'<1, LOD<2. r^2 ^values are represented as black for r^2 ^= 1, white for r^2 ^= 0, with intermediate values for 0<r^2 ^< 1 indicated by shades of grey. The numbers within the squares represent the D' or r^2 ^scores for pairwise LD.

Table 2 presents the results of the case-control logistic regression analysis. SNP rs2854386 at *CXCR1 *was associated with susceptibility to CL, but not to ML, when each of these patient groups was compared with either the NC, the DTH^+^, or the combined NC+DTH^+ ^control groups. CL is associated (OR 2.38; 95% CI 1.19-3.40; global *P *= 0.006) with the common C allele. Analysis for susceptibility to *L. braziliensis per se *(i.e. CL+ML compared to NC+DTH^+^) did not improve the significance (Table 2) suggesting that, although CL disease usually precedes ML disease, there was something different about the ML patient group which meant that they did not contribute to this association. Similarly, significance observed at rs2854386 for CL disease under a dominant model in the FBAT analysis (Table 3) was not improved when the data were analysed for susceptibility to *L. braziliensis per se*. CL disease was associated with the common C allele. Interestingly, in this analysis, transmission disequilibrium of alleles from heterozygous parents to ML disease patients only (Table 3) showed disease associated with the opposite G allele at rs2854386, suggesting opposing influences on the role of this gene in CL versus ML disease. This was principally determined by disease associated with the dominant G allele in heterozygotes (FBAT Z score 2.221; *P *= 0.026) in the genotype analysis, with the homozygous recessive CC genotype being protective for ML (FBAT Z score = -1.999; *P *= 0.04). This was measurable in the family-based analysis but the genotype-wise test (not shown) was not valid in the case-control analysis due to the smaller sample size. Similar but weaker allele-wise associations were observed in the case-control analysis for SNP *CXCR1*_rs2234671 (Table 2), which is in strong (but not complete) linkage disequilibrium with *CXCR1*_rs2854386 in the family founders (Figure 1) and unrelated NC controls (data not shown) in our study.

**Table 2:** Results of logistic regression analysis

Gene/Marker	Groups Compared	Allele	OR	95% CI	*Global P *value
CXCR2_rs4674259	CL vs DTH	C vs T	1.03	0.60-1.76	0.908

	CL vs NC	C vs T	0.91	0.53-1.58	0.752

	ML vs DTH	C vs T	1.92	1.05-3.51	**0.028**

	ML vs NC	C vs T	1.71	0.93-3.14	0.076

	CL vs DTH+NC	C vs T	0.97	0.60-1.56	0.909

	ML vs DTH+NC	C vs T	1.82	1.07-3.07	**0.023**

	CL+ML vs DTH+NC	C vs T	1.26	0.85-1.88	0.239

CXCR1_rs2854386	CL vs DTH	C vs G	2.31	1.07-4.96	**0.027**

	CL vs NC	C vs G	2.48	1.21-5.10	**0.009**

	ML vs DTH	C vs G	1.55	0.72-3.35	0.254

	ML vs NC	C vs G	1.72	0.84-3.54	0.127

	CL vs DTH+NC	C vs G	2.38	1.23-4.57	**0.006**

	ML vs DTH+NC	C vs G	1.63	0.84-3.14	0.132

	CL+ML vs DTH+NC	C vs G	2.01	1.19-3.40	**0.007**

CXCR1_rs2234671	CL vs DTH	C vs G	2.06	0.93-4.56	0.069

	CL vs NC	C vs G	1.91	0.89-4.10	0.087

	ML vs DTH	C vs G	1.73	0.74-4.01	0.190

	ML vs NC	C vs G	1.62	0.72-3.63	0.226

	CL vs DTH+NC	C vs G	1.96	0.98-3.89	**0.044**

	ML vs DTH+NC	C vs G	1.66	0.79-3.46	0.160

	CL+ML vs DTH+NC	C vs G	1.84	1.04-3.23	**0.030**

SLC11A1_rs 17235416	CL vs DTH	DEL vs IN	1.35	0.63-2.87	0.428

	CL vs NC	DEL vs IN	0.87	0.42-1.79	0.714

	ML vs DTH	DEL vs IN	1.10	0.53-2.30	0.781

	ML vs NC	DEL vs IN	0.73	0.35-1.48	0.383

	CL vs DTH+NC	DEL vs IN	1.07	0.57-1.99	0.820

	ML vs DTH+NC	DEL vs IN	0.88	0.47-1.66	0.709

	CL+ML vs DTH+NC	DEL vs IN	0.97	0.58-1.62	0.929

Under an additive model for case-control comparisons of CL, ML and *L. braziliensis per se *(CL+ML) disease groups with NC, DTH^+ ^or NC+DTH^+ ^control groups. Only the data for markers where significant associations (P = 0.05; bold) were observed in this or the family-based (Table 3) analysis are shown. OR = odds ratio; CI = 95% confidence interval.

**Table 3:** FBAT analysis of family data

Gene/Marker	Phenotype	Model	Allele/Genotype	Allele/Genotype Frequency	# Families	Obs T	Exp T	Z score	*P *value
CXCR1_rs2854386	CL	Dominant	C	0.83	12	35	29	2.002	**0.045**

	ML	Dominant	G	0.17	20	17	12	1.999	**0.046**

	ML	Genotype	CC	0.69	20	7	12	-1.999	**0.046**

	ML	Genotype	CG^1^	0.28	22	18	12	2.221	**0.026**

	CL+ML	Dominant	C	0.83	12	35	29	2.002	**0.045**

CXCR1_rs2234671	CL	Dominant	C	0.85	12	36	32	1.563	0.118

	ML	Dominant	G	0.15	18	14	11	1.604	0.108

	ML	Genotype	CG	0.30	19	14	9	1.964	**0.049**

	CL+ML	Dominant	C	0.85	12	36	32	1.563	0.118

SLC11A1_rs 17235416	CL	Additive	IN	0.86	27	98	88	2.549	**0.011**

	ML	Additive	IN	0.86	21	32	32	0.209	0.834

	CL+ML	Additive	IN	0.86	28	105	95	2.333	**0.020**

	CL	Dominant	DEL	0.14	24	30	12	-2.198	**0.028**

	ML	Dominant	DEL	0.14	19	9	10	-0.426	0.670

	CL+ML	Dominant	DEL	0.14	25	25	32	-2.179	**0.029**

	CL	Genotype	IN/IN	0.75	24	35	27	2.198	**0.028**

	CL	Genotype	IN/DEL^2^	0.23	27	28	33	-1.206	0.228

	ML	Genotype	IN/IN	0.75	19	12	11	0.426	0.670

	ML	Genotype	IN/DEL^2^	0.23	21	8	10	-1.021	0.307

	CL+ML	Genotype	IN/IN	0.75	25	38	30	2.179	**0.029**

	CL+ML	Genotype	IN/DEL^2^	0.23	28	29	34	-1.409	0.159

FBAT analysis for transmission of alleles from heterozygous parents to CL, ML and *L. braziliensis per se *(CL and ML) individuals in families. # families = number of families informative for the FBAT analysis. A positive Z score indicates association with disease; a negative Z score indicates the non-associated or protective allele or genotype. Obs T = observed transmissions; Exp T = expected transmissions. ^1^Insufficient informative families (# = 4) for the GG genotype (frequency 0.03) to contribute to the analysis; ^2^Insufficient informative families (# = 5,4,5) for the DEL/DEL genotype (frequency 0.02) to contribute to the analysis for CL, ML or CL+ML.

*CXCR1*_rs2234671 did not achieve significance for CL disease in the FBAT analysis (Table 3), but the presence of the G allele in the heterozygous genotype conferred disease association for ML (FBAT score 1.964; *P *= 0.049). Haplotype analysis (Table 4) across the 3 *CXCR2/CXCR1 *markers rs467259_rs2854386_rs2234671 confirmed that the common haplotype T_C_C (frequency 0.47) was significantly over-transmitted to individuals in the families with CL only (χ^2^_1df _= 6.62; *P *= 0.01) and significantly under-transmitted to individuals in the families with ML (χ^2^_1df _= 4.42; *P *= 0.04), consistent with the case-control analysis (Table 2) that showed ML associated with the rarer C allele at *CXCR2*_rs4674259 (OR = 1.82; 95% CI 1.07-3.07; global *P *= 0.023). Inclusion of the *CXCR2 *SNP rs4674259 was necessary to observe the haplotype associations, which were not explained by over- or under-transmission of the shorter C_C haplotype for rs2854386_rs2234671 (Table 4). The opposing effects of polymorphism at *CXCR1/2 *on CL and ML was clearly neutralized in analysis of haplotype transmission to the combined CL+ML group (Table 4).

**Table 4:** Haplotype analysis of family data

Disease Phenotype	Haplotypes	Haplotype Frequency	Over/Under Transmitted	rs4674259	rs2854386	rs2234671
CL	**T_C**	0.47	Over	O = 160; E = 145; χ^2^_1df _= 5.88; ***P *= 0.015**		

	**C_C**	0.16	-		O = 62; E = 67; χ^2^_1df _= 2.02; NS	

	**T_C_C**	0.47	Over	O = 152; E = 136; χ^2^_1df _= 6.62; ***P *= 0.010**		

ML	**T_C**	0.47	Under	O = 41; E = 49; χ^2^_1df _= 5.19; ***P *= 0.023**		

	**C_C**	0.16	-		O = 17; E = 15; χ^2^_1df _= 0.08; NS	

	**T_C_C**	0.47	Under	O = 39; E = 46; χ^2^_1df _= 4.22; ***P *= 0.040**		

CL or ML	**T_C**	0.47	-	O = 201; E = 194; χ^2^_1df _= 0.96; NS		

	**C_C**	0.16	-		O = 80; E = 82; χ^2^_1df _= 1.36; NS	

	**T_C_C**	0.47	-	O = 190; E = 181; χ^2^_1df _= 1.59; NS		

TRANSMIT haplotype association analysis for SNPs rs4674259_rs2854386_rs2234671 across *CXCR2_CXCR1 *showing over- and under-transmission for the common haplotypes in families analysed for the CL only phenotype, ML only phenotype, and CL or ML. O = observed number of transmissions; E = expected number of transmissions.

At *SLC11A1*, none of the polymorphisms showed association with either CL or ML disease in the case-control logistic regression analysis (Table 2). Association between the *SLC11A1 *1729+55del4 [21] IN/DEL and CL (FBAT Z score +2.198 for the IN/IN homozygous genotype, *P *= 0.028) but not ML disease was observed in the family-based analysis (Table 3), again with the ML group not apparently contributing to the association. The disease allele is the common insertion allele, which is recessive, while the deletion is protective under the dominant model. Given the low level of linkage disequilibrium between the 1729+55del4 IN/DEL and rs2854386 (Figure 1), the associations observed between CL and *SLC11A1 *and between CL and *CXCR1 *are likely to be independent. There was insufficient power in the sample to determine whether these two loci had independent effects, or whether there was any interaction between them.

## Discussion

The study presented here has provided interesting preliminary data which support roles for both *CXCR1 *and *SLC11A1 *in determining the outcome of *L. braziliensis *infection. Although the FBAT analysis supported the case-control analysis for *CXCR1*, the power of our study was limited by small sample size and MAF < 0.2 for the markers (rs2854386, rs2234671) of particular interest. In the context of the admixed population found in Brazil, the family-based analysis provides some confidence that the results are not due to mismatch between case and control groups. For that reason, we also have some confidence in the association between *SLC11A1 *observed in the more powerful family-based analysis for CL disease, even though this was not replicated in the case-control analysis. Further replication studies in larger cohorts will be essential to validate our results. Nevertheless, the data provide interesting insight into the possible roles of PMN and macrophages in leishmaniasis caused by *L. braziliensis*.

At present we do not know the functional basis to the association between *CXCR1 *rs2854386 and CL versus ML disease. This SNP lies in the 3' region of the *CXCR1 *gene, adjacent to rs4674259 in the 5' region of the *CXCR2 *gene. Opposing over- and under-transmitted associations with the common ancestral T_C_C haplotypes for CL and ML, but not the shorter C_C haplotypes, suggests location of the functional variant in the regulatory region between the genes that could affect expression of either of them. Although *CXCR1 *rs2854386 is in strong linkage disequilibrium with rs2234671, which encodes a mis-sense mutation that alters the amino acid sequence from Ser-to-Thr at position 276 of the protein, the haplotype analysis did not support over- and under-transmission of the C_C haplotype between these two markers as the reason for the opposing associations. Interestingly, the allele frequencies for the minor allele (MAF) for rs2854386 (0.218) and rs2234671 (0.194) observed in Brazil were high compared to those observed in Caucasian (CEU) (0.058 for both) and Asian (JPT) populations (0.100 for both), perhaps reflecting the contribution of African haplotypes (e.g. YRI MAFs 0.425 and 0.317, respectively) to the Brazilian population near Salvador, a city with a significant population of African origin. Failure to see complete concordance between the results at these two markers in our analysis could be due to differences in genotyping success between individuals, or to the presence of African haplotypes. Association of CL with the common ancestral T_C_C haplotype suggests that disease is associated with a fully functional variant, and hence that influx of PMN is associated with CL disease. This is consistent with data [6] and hypotheses [7] relating to the possible role of PMN in initiation of a lesion following the delivery of parasites via the sand fly bite. Association of ML with the rare derived G allele, which we assume to be the functionally compromised allele/haplotype, suggests that PMN may have an important positive role to play in preventing this form of the disease. Recent studies have shown, for example, that PMN can play an important role in initiating and regulating innate immune defences that protect mucosal surfaces from fungal infection [22]. Failure to protect the mucosa from early invasion and injury could result in local presentation of antigen and amplification of the acquired T cell-mediated proinflammatory response that is associated with ML disease.

The association of CL disease with the common 1729+55del4 IN variant at *SLC11A1 *is also of interest in relation to the putative role of this molecule, which is expressed in mature macrophages [23], dendritic cells [24] and PMN [25], in regulating expression of secretory leukocyte protease inhibitor and hence affecting the wound healing response [12]. Differences in lesion development have not been observed following subcutaneous needle injection of either *L. major *[26] or *L. mexicana *[27] into *Slc11a1 *congenic mice, suggesting that the genetic influence of *SLC11A1 *on susceptibility to CL following natural infection in humans might be mediated by the effect on the wound healing response to the sand fly bite. This means that the mechanism by which *SLC11A1 *influences CL disease may be different to its influence on visceral leishmaniasis in mice following intravenous needle injection [28], or in natural infection of dogs [29,30] and humans [31,32], consistent with its many pleiotropic effects [11]. Our study was not sufficiently powered to look for interaction between the *CXCR1 *and *SLC11A1 *in this study, and further work will be needed to determine whether the association at *SLC11A1 *relates to its role in PMN, macrophages or dendritic cells at the site of infection. For the present, our study begins to provide novel insight to the possible role of PMN in lesion development of leishmaniasis caused by *L. braziliensis *infection in Brazil.

## Conclusions

The study supports roles for *CXCR1 (IL8RA) *and *SLC11A1 *in the outcome of *L. braziliensis *infection in humans. Previous data in mice showing that Slc11a1 does not influence cutaneous lesion development following needle injection of *Leishmania *suggests that its role here might relate to the action of PMN, macrophage and/or dendritic cells in the wound healing response to the sand fly bite. Together with the *CXCR1 *association, the data are consistent with hypotheses relating to the possible role of PMN in initiation of a lesion following the delivery of parasites via the sand fly bite. Association of ML with the rare derived G allele at *CXCR1 *rs2854386 suggests that PMN also have an important positive role to play in preventing this form of the disease.

## Competing interests

The authors declare that they have no competing interests.

## Authors' contributions

LC carried out the field collection and preparation of the samples, performed the genotyping, and participated in the statistical analysis and interpretation of the data. SEJ and ENM trained LC in the laboratory for genotyping techniques, in database entry and use of the genetic database GenIE in Cambridge, and in genetic statistical analysis methods. SEJ cross-checked statistical analyses and carried out additional statistical tests. EM, JO, AM and LHG participated in the field collection of data, processing of DNA samples and database entry in Brazil. ML is the doctor responsible for confirmation of the ML cases by performing ENT exams. ARJ trained the field group, initial selection of cases from the health post, assisted with field collection of data and participated in the design of the study. EMC helped conceive the study, initial selection of cases from the health post, and provided the logistical support to make the study possible. JMB participated in the design of the study, conceived the specific hypothesis to be tested, made the final interpretation of the data, and prepared the manuscript. All authors read and approved the final manuscript.

## Pre-publication history

The pre-publication history for this paper can be accessed here:

http://www.biomedcentral.com/1471-2350/11/10/prepub
